# Use of intravenous lipid emulsions in drug-induced toxicities: a 2025 narrative review

**DOI:** 10.1186/s13613-025-01601-5

**Published:** 2025-11-17

**Authors:** Gauthier Nendumba, Sydney Blackman, Nathan De Lissnyder, Marine Cillis, Patrick M. Honore

**Affiliations:** 1 CHU UCL Namur Godinne, UCL University, Campus Godinne, Yvoir, Belgium; 2 CHIREC Hospitals, ULB University, Brussels, Belgium; 3 Universitair Ziekenhuis Brussel, VUB University, Brussels, Belgium; 4 Faculty of Medicine, Experimental Research Laboratory Institute of the Catholic Louvain Medical School, Brussels, Belgium; 5 CHU UCL Godinne Namur, UCL Louvain Medical School, Campus Godinne Avenue G Thérasse 1, Yvoir, 5530 Namur Belgium

**Keywords:** Intravenous lipid emulsions, Lipophilic poisoning, Bupivacaine, Lipid sink, Toxicity management, Antidote

## Abstract

Intravenous lipid emulsions (ILE) were first proposed in 1998 as a treatment for bupivacaine-induced cardiac arrest. Since then, their use has expanded to include poisonings by various lipophilic drugs such as tricyclic antidepressants, calcium channel blockers, and antipsychotics. This 2025 narrative review explores the evolving pathophysiological mechanisms of ILE therapy, including the lipid sink and lipid shuttle theories, as well as non-scavenging cardiotonic effects such as membrane stabilization, mitochondrial support, and modulation of vascular tone. It summarizes recent findings from randomized controlled trials, cohort studies, animal models, and case registries. While clinical trials demonstrate potential benefits—particularly in tramadol, clozapine, and organophosphate poisonings—mortality reduction remains unproven, and evidence is limited by study heterogeneity and low methodological quality. Adverse effects, although rare, include acute pancreatitis, interference with laboratory testing, and fat overload syndrome, especially at high infusion volumes. Current guidelines recommend ILEs as a first-line treatment for local anesthetic systemic toxicity and as a second-line option in life-threatening poisonings involving other lipophilic agents. However, significant uncertainty remains regarding optimal indications, dosing strategies, and long-term safety. High-quality, multicenter studies and updated registries are needed to refine these recommendations and clarify the role of ILEs in clinical toxicology.

## Introduction

 Intravenous lipid emulsions (ILE) have been used for decades in parenteral nutrition to provide additional calories and essential fatty acids. They also serve as carriers for lipophilic drugs, with propofol being the most notable example. ILEs are composed of fine triglyceride droplets stabilized by phospholipid surfactants, with an average particle size ranging from 200 to 600 nm. These emulsifiers, which are both lipophilic and hydrophilic, encapsulate the lipid droplets. Sodium hydroxide is used to adjust the pH, while glycerin ensures appropriate osmolarity. The final mixture is administered intravenously [1].

In 1979, Albright GA first drew attention to the role of local anesthetics—such as bupivacaine and etidocaine—in refractory cardiac arrest unresponsive to conventional resuscitation techniques [2]. The potential therapeutic application of ILEs for bupivacaine toxicity was first demonstrated in an animal model by Weinberg et al. in 1998 [3]. A few years later, Rosenblatt et al. published the first clinical case reporting successful use of ILEs in a patient who experienced cardiac arrest following presumed bupivacaine and mepivacaine toxicity [4]. This case marked a turning point in the use of ILEs, particularly in the management of local anesthetic systemic toxicity (LAST).

Since then, the clinical scope of ILE therapy has expanded beyond LAST. Evidence—primarily derived from animal models and clinical case reports—suggests that ILEs may be an effective antidote in cases of cardiotoxicity or coma induced by a range of lipophilic agents, including tricyclic antidepressants, verapamil, propranolol, thiopental, bupropion, lamotrigine, sertraline, trazodone, and quetiapine [5].

For instance, in 2008, Sirianni et al. reported the case of a 17-year-old female who developed seizures and cardiovascular collapse after ingesting approximately 7.95 g of bupropion and 4 g of lamotrigine. Despite 70 min of conventional cardiopulmonary resuscitation, sustained circulation could not be achieved. Following the administration of a 100 mL intravenous bolus of 20% lipid emulsion, a stable and effective pulse was observed within 1 min. Although the patient subsequently developed significant acute lung injury, she experienced rapid cardiovascular recovery and was ultimately discharged with near-normal neurological function [6].

Since 2009, there has been a marked increase in case reports documenting ILE use for intoxications involving lipophilic agents, accompanied by several literature reviews [5]. Notably, a prospective multicenter study conducted in New Zealand between August 2009 and August 2012 established a registry to collect data on ILE use in poisoning cases [7]. Known as the LIPAEMIC study, this registry enrolled 48 patients, 38 of whom had been exposed to agents other than long-acting local anesthetics.

However, the overall quality of available data remains limited due to the paucity of randomized controlled trials. As a result, recommendations from authoritative bodies—including the European Resuscitation Council (ERC), the American Academy of Clinical Toxicology (AACT), and the American Heart Association (AHA)—are based on low levels of evidence. In this review, we aim to summarize the current literature and provide an updated perspective on the role of ILEs in the management of xenobiotic toxicity as of 2025.

## Methodology

This narrative review was conducted to synthesize and critically evaluate the available evidence on the use of ILEs in the management of xenobiotic toxicity. A structured literature search was performed in PubMed, covering publications from 1998 through April 2024. The search strategy combined the terms “lipid emulsion,” “intravenous lipid therapy,” “lipophilic intoxication,” “toxicity,” and “overdose,” using Boolean operators (AND, OR, NOT) to optimize the identification of relevant articles.

We included clinical studies (randomized controlled trials, cohort studies, case series), animal studies, and systematic reviews published in English. Selected case reports were also retained when they offered meaningful clinical insights or supported emerging hypotheses regarding the mechanisms of action of ILEs. In addition, guidelines and position statements from major toxicology organizations (e.g. American Academy of Clinical Toxicology, American College of Medical Toxicology, American Heart Association, European Resuscitation Council) were reviewed.

Titles and abstracts were screened for relevance, followed by full-text evaluation of eligible articles. Given the narrative nature of this review and the heterogeneity of the included literature, no formal quality assessment or risk-of-bias scoring was performed. The objective of this review is to provide a comprehensive and current overview of the efficacy, mechanisms, and safety profile of ILEs in the treatment of drug-induced toxicity.

## Pathophysiology

The mechanism by which ILEs reverse xenobiotic overdose remains an area of active investigation and debate. The most widely accepted hypothesis suggests that ILE therapy exerts a multimodal resuscitative effect, involving both scavenging and non-scavenging mechanisms [8].

Historically, the first proposed mechanism was the Lipid Sink Theory, introduced by Weinberg et al. in 1998 following experimental studies in rats poisoned with bupivacaine and treated with lipid emulsions. Their findings demonstrated that ILE infusion altered the bupivacaine dose required to induce cardiac arrest—an effect partially attributed to sequestration of the drug into a “lipid sink” [3]. According to this theory, ILEs create an intravascular lipid phase capable of binding lipophilic compounds, thereby reducing the free (active) concentration of the toxic agent in plasma [9].

This theory has since evolved into the more comprehensive Lipid Shuttle Theory, which posits that beyond sequestration, the lipid phase facilitates redistribution of lipophilic xenobiotics to less sensitive compartments. Laboratory studies have shown that while ILEs reduce the elimination half-life of bupivacaine and its concentrations in the brain and heart, they simultaneously increase the distribution half-life and hepatic concentrations of the drug. These findings suggest ILE-induced acceleration of drug redistribution and clearance. Specifically, ILE administration results in decreased bupivacaine levels in the frontal lobe and cerebellum, reduced organ-to-blood partitioning in the heart, brain, lungs, and kidneys, and increased hepatic uptake and decay rates in critical organs. The proposed mechanism involves the formation of multiple lipid compartments in circulation that absorb highly lipophilic drugs from high-perfusion organs such as the brain and heart, enabling subsequent redistribution to the liver, adipose tissue, or muscle for detoxification and storage. This redistribution is thought to be a key factor in successful ILE-based resuscitation, particularly for compounds with high lipid solubility [10].

An illustrative example of this phenomenon was published by Nendumba et al., who reported a case involving a 49-year-old man admitted 3 h after intentional ingestion of 5000 mg of immediate-release trazodone. His Glasgow Coma Scale (GCS) score fluctuated between 11 and 13/15. A 20% lipid emulsion (Intralipid, Fresenius Kabi, India) was administered intravenously as an initial bolus of 1.5 mL/kg, followed by a continuous infusion of 0.25 mL/kg/min over 30 min. Plasma trazodone concentration measured 8 h post-ingestion was 3678 ng/mL. Four hours later—30 min after the end of ILE infusion—the concentration had dropped sharply to 111.6 ng/mL, but rebounded to 4784 ng/mL 1 h later. The authors hypothesized that this rebound reflected redistribution from tissue stores or from the lipid compartment (given that the half-life of ILE is shorter than that of trazodone) [11].

**Fig. 1:**
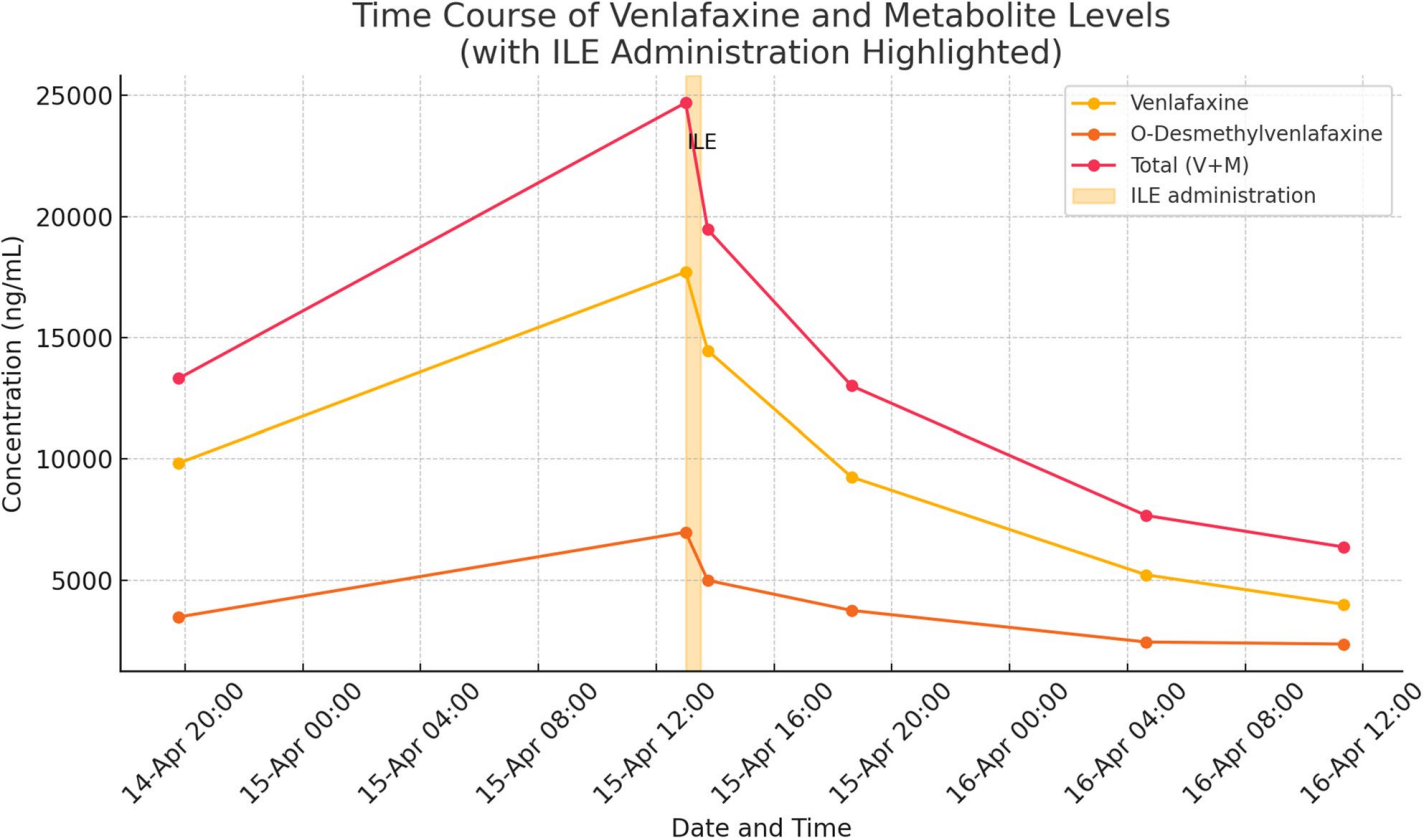
Time course of venlafaxine and metabolite levels. Personal data

At our own institution, we recently admitted a patient to the ICU following intentional venlafaxine overdose. The patient presented with seizures, severely altered mental status (GCS 5/15), and QRS widening. We measured venlafaxine and metabolite levels before and after ILE administration. As shown in Fig. 1, both venlafaxine and its metabolite levels decreased significantly during and after ILE infusion—this time without a rebound effect. This observation may be attributed to specific pharmacokinetic properties of venlafaxine, such as its elimination half-life, although further analysis is warranted.

Nevertheless, lipophilicity does not appear to be the sole determinant of ILE efficacy. A pivotal study by Levine et al. investigated whether survival following ILE treatment was associated with the lipophilicity of the ingested xenobiotic. Conducted between May 2012 and December 2018, the study analyzed 134 cases recorded in the Toxicology Investigators Consortium’s lipid sub-registry. Among these, 108 patients (80.6%) survived. However, no statistically significant relationship was found between survival and the lipophilicity of the intoxicant, as measured by the partition coefficient (log p) (linear model: *p* = 0.89; polynomial model: *p* = 0.10). While this study does not entirely exclude a role for lipophilicity, it raises important questions about its centrality in the mechanism of ILE action [12].

Indeed, growing evidence suggests that beyond lipid-scavenging capacity, ILEs also exert direct cardiotonic effects, demonstrated in both preclinical and clinical settings. These effects are mediated through several distinct physiological mechanisms. Notably, ILE administration alone has been shown to enhance the maximal rates of left intraventricular pressure rise and fall, as well as the rate–pressure product [10–13].

**Fig. 2:**
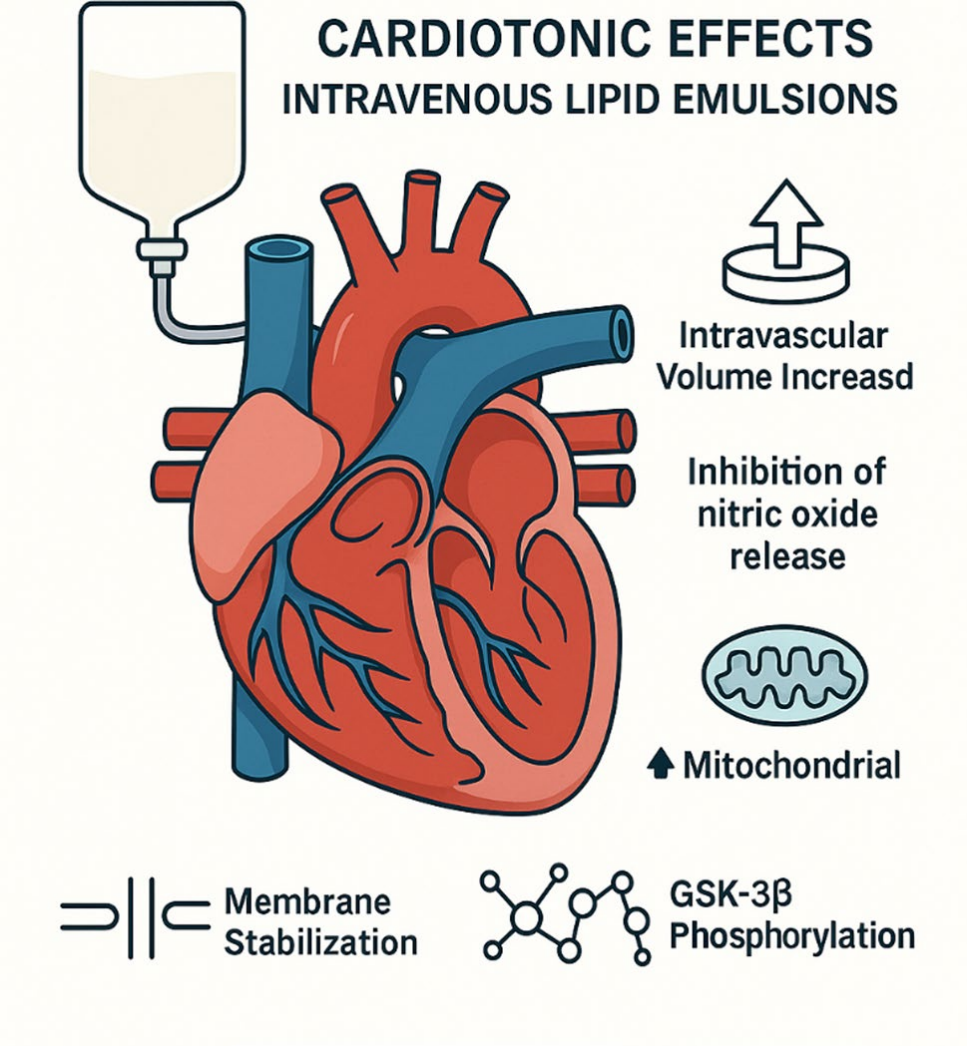
Cardiotonic effects intravenous lipid emulsions

1. Improved cardiac output and preload: ILEs may augment cardiac contractility, cardiac output, and preload via expansion of intravascular volume. However, volume expansion alone is unlikely to account for all effects, as ILEs have been shown to outperform saline in improving hemodynamic parameters.
2. Inhibition of nitric oxide (NO)-mediated vasodilation: ILEs may contribute to hemodynamic stabilization by inhibiting the release of endothelial-derived NO. The polyunsaturated fatty acid “linolenic acid”, a component of both Intralipid and Lipofundin MCT/LCT, has been shown to attenuate acetylcholine-induced, NO-mediated vasodilation. Consequently, ILEs may increase arterial blood pressure and systemic vascular resistance, albeit at the potential cost of reduced vascular compliance and impaired flow-mediated vasodilation [14].
3. Support of mitochondrial function: ILEs may enhance mitochondrial function by increasing fatty acid supply and mitigating toxin-induced mitochondrial dysfunction. In particular, they appear to counteract inhibition of carnitine-acylcarnitine translocase—an enzyme essential for transporting long-chain fatty acids into cardiac mitochondria—thereby supporting adenosine triphosphate (ATP) production via β-oxidation. Cardiotoxic agents such as chloroquine, propranolol, and doxorubicin impair mitochondrial function and ATP synthesis through excessive production of reactive oxygen species (ROS); ILEs have demonstrated the ability to reduce ROS generation and preserve mitochondrial integrity in such contexts.
4. Stabilization of cellular membranes and modulation of ion channels: ILEs may help reverse sodium channel blockade induced by lipophilic toxins and enhance intracellular calcium concentrations via direct activation of calcium channels. The resulting increase in calcium influx contributes to both positive inotropic (increased myocardial contractility) and chronotropic (increased heart rate) effects.
5. Modulation of intracellular signaling pathways: ILEs may induce phosphorylation of glycogen synthase kinase-3β (GSK-3β). In experimental models of ischemia–reperfusion injury, post-ischemic administration of lipid emulsions has been associated with reduced myocardial damage, likely through inhibition of mitochondrial permeability transition pore opening mediated by GSK-3β phosphorylation (Fig. 2).

Taken together, these mechanisms not only enhance cardiac output but also facilitate toxin redistribution—a process described by the lipid shuttle model [1].

From these hypotheses and findings, it is evident that lipophilicity is only one of several contributing factors in the complex, multimodal mechanism of action of ILEs. Moreover, clinical observations suggest that the benefits of ILE therapy may extend beyond sequestration of lipophilic toxins, with notable effects on inotropy, chronotropy, and preload. Unfortunately, in critically ill patients, definitive conclusions regarding survival benefit remain elusive due to the lack of appropriate control groups in existing studies. Nonetheless, these findings point to a broader therapeutic potential for ILEs and underscore the need for further high-quality research [8].

## Animal studies

Conducting large randomized clinical trials to assess the efficacy of ILEs as an adjunctive treatment for cardiovascular collapse induced by non-local anesthetic toxicants remains extremely challenging. Major obstacles include achieving adequate sample sizes, standardizing patient selection criteria, ensuring methodological consistency across study sites, and minimizing bias—all within a hemodynamically unstable target population.

Given these limitations, animal models represent a valuable alternative for preclinical investigation. In 2020, Fettiplace et al. published a systematic review and meta-analysis evaluating the effect of ILE therapy on mortality in animal models of drug-induced cardiac toxicity. The primary outcome was overall mortality, assessed using a random-effects model due to inter-study heterogeneity. Secondary analyses examined subgroups, including local anesthetic systemic toxicity, non-local anesthetic toxicity, swine-based models, and non-swine models (e.g., rat, rabbit, dog).

A total of 58 studies were included. Across all models, ILE treatment significantly reduced mortality, with a pooled odds ratio (OR) for death of 0.26 (95% CI 0.16–0.44; Z = 5.21; *p* < 0.00001; Cohen’s *d* = 0.72; *n* = 60). Subgroup analysis confirmed reduced mortality in both local anesthetic toxicity models (OR: 0.16; 95% CI 0.10–0.33) and non-local anesthetic models (OR: 0.43; 95% CI 0.22–0.83).

Observed heterogeneity (Cochran’s *Q* = 132; *df* = 59; *p* < 0.01; *I²* = 55%) was primarily attributable to the type of animal model used. When stratified by species, heterogeneity decreased markedly (*I²* ≤ 12%). Notably, swine models demonstrated benefit only in local anesthetic toxicity (OR: 0.28; 95% CI 0.11–0.70; *p* = 0.0033), whereas non-swine models showed consistent benefit across all toxicants (OR: 0.10; 95% CI 0.06–0.16; *p* < 0.00001). The lack of efficacy observed in swine exposed to non-local anesthetics may be attributable to factors such as dosing protocol, experimental design, or species-specific hypersensitivity reactions to lipid emulsions [15].

## Review of recent data

### Meta-analysis

A meta-analysis conducted by Yu et al. in 2018 examined the use of ILEs in cases of organophosphate (OP) poisoning—a significant public health concern in South Asia, particularly among farming populations [11]. OP poisoning remains a major cause of morbidity and mortality in these regions, with case fatality rates ranging from 10 to 40%, despite standard treatments such as anticholinergic agents, oximes, and best supportive care.

The meta-analysis included seven randomized controlled trials (RCT), comprising a total of 630 patients who met the inclusion criteria. The pooled data demonstrated a significantly higher cure rate in patients treated with lipid resuscitation compared to those in the control group (OR = 2.54, 95% CI [1.33, 4.86]). Additionally, four studies provided mortality data from 301 patients, showing that lipid resuscitation significantly reduced mortality in OP poisoning cases compared to controls (OR = 0.31, 95% CI [0.13, 0.74]; *p* = 0.009).

The authors concluded that initiating ILE therapy upon arrival of an OP-poisoned patient in the emergency department may improve overall prognosis and enhance specific organ functions, such as liver enzyme levels.

However, several limitations were noted. The methodological quality of the included studies was relatively low, with missing information on blinding procedures. Furthermore, all seven RCTs were conducted in a single country (China), limiting the generalizability of the findings. Four of the seven studies focused exclusively on patients with severe OP poisoning, yet baseline characteristics—such as respiratory and circulatory function—were not reported.

Additionally, the studies did not address key complications associated with lipid resuscitation, such as acute pancreatitis and lipid embolism, making it difficult to fully evaluate the safety of this therapeutic approach. These gaps underscore the need for further high-quality, multicenter trials to establish the efficacy and safety of ILE therapy in OP poisoning [16].

### Randomized controlled trials

Several RCTs have explored the efficacy of ILEs in acute poisonings unrelated to local anesthetics. These studies are presented below in approximate chronological order.

Majeed et al. (conducted between November 2012 and October 2014) evaluated 100 patients with confirmed OP poisoning. Participants were randomized to receive either standard treatment with atropine and pralidoxime or atropine in combination with ILEs. The ILE group demonstrated significant improvements in GCS scores and mean arterial pressure (MAP), along with reduced ventilation requirements (*p* < 0.006), shorter ventilation duration (*p* < 0.002), reduced ICU length of stay (*p* < 0.049), and lower mortality rates (*p* < 0.05). However, the study did not report on adverse effects related to ILE administration [17].

Taftachi et al. [18] conducted one of the earliest RCTs in this context, enrolling 30 patients with non-local anesthetic poisoning and a GCS score ≤ 9. Patients were randomized to receive either ILEs or standard care. The intervention group showed a statistically significant, though clinically modest, improvement in GCS (mean increase of 3 points versus 2 points in controls after 6 h). No differences were observed in extubation time, respiratory or hemodynamic parameters, and no adverse effects were reported [18].

Elabdeen et al. (December 2016 to November 2017) assessed the use of ILEs as adjuvant therapy in 50 patients with acute aluminum phosphide poisoning. Although the difference in mortality between the intervention and control groups was not statistically significant (56% vs. 76%), the need for intubation and mechanical ventilation was significantly reduced (36% vs. 92%). The authors concluded that ILE may be effective and safe in this context [19].

Kazemifar et al. (2017–2018) investigated the use of ILEs in 80 patients with tramadol intoxication. The primary endpoint was the occurrence of tonic-clonic seizures during hospitalization. None of the patients in the intervention group experienced seizures, compared to 15 in the control group (*p* < 0.001). However, potential adverse effects of ILEs were not assessed [20].

Behnoush et al. (April 2019 to February 2021) conducted a trial including 120 patients with isolated tramadol poisoning and GCS ≤ 12. Patients were randomized to receive either ILEs or 0.9% saline. The ILE group showed significantly higher post-treatment GCS scores (12 vs. 10; *p* = 0.03), shorter hospital stays (2 days vs. 4 days; *p* < 0.01), and lower seizure incidence (16/60 vs. 30/60; *p* < 0.01). No adverse effects were reported [21].

Basiouny et al. (March 2019 to February 2020) evaluated 40 patients with moderate to severe antipsychotic poisoning, with clozapine being the most common agent. Compared to controls, the intervention group exhibited significantly higher GCS scores at both 6 h (15 vs. 10) and 12 h (15 vs. 14) post-admission. ECG normalization was more frequent (80% vs. 40%), QTc prolongation was reduced (20% vs. 55%), and ICU admissions and intubation rates were lower (5% vs. 15%). No adverse effects were observed [22].

Elgazzar et al. (December 2019 to May 2020) focused specifically on clozapine poisoning, enrolling 40 patients who received either standard care or SMOF lipid emulsion. The ILE group had significantly higher GCS scores at 6 h (13.1 ± 2.3 vs. 9.2 ± 2.0; *p* < 0.001) and 12 h (14.3 ± 1.5 vs. 9.6 ± 2.0; *p* < 0.001), less QTc prolongation (5% vs. 45%; *p* = 0.003), and shorter hospital stays (18 vs. 71 h; *p* < 0.001). No major adverse effects were reported [23].

Pannu et al. [24] conducted an open-label RCT on 45 patients with OP poisoning to assess whether ILEs reduced the required dose of atropine. No significant differences were observed between the intervention and control groups regarding atropine use, hemodynamic variables, hospital stay, or mortality. The study concluded that ILEs did not confer additional clinical benefits in this context, and no adverse effects were reported [24].

### Prospective cohort studies

An open-label prospective cohort pilot study, conducted in 2019 by Chhabria et al., aimed to assess the safety of lipid emulsions in OP poisoning. Patients with symptomatic OP poisoning who met the inclusion and exclusion criteria were treated with 100 mL of 20% ILE, administered alongside the standard of care, after obtaining informed consent. A total of 40 patients were enrolled in the study group. Outcomes such as morbidity, mortality, and adverse effects were compared with a historical control group. Although no significant reduction in mortality was observed, the study reported a shorter duration of mechanical ventilation and hospital stay, along with earlier resolution of hypernatremia in the intervention group [25].

Another study conducted by Gil et al. from 2010 to 2012, explored the use of ILEs in cases of intentional glyphosate poisoning—a herbicide widely used in agriculture. The study included 64 patients, 22 of whom were treated with ILE. The control group consisted of 42 patients matched based on the ingested volume of glyphosate and the time of hospital arrival. Age, sex, the amount of ingestion, and time lag to the emergency room were comparable in both groups. The results showed significantly lower incidences of hypotension (0% vs. 40%, *p* = 0.0001) and arrhythmias (0% vs. 22.7%, *p* = 0.048) in the intervention group. The authors suggested that ILEs could mitigate toxicity caused by the partially lipophilic surfactant present in glyphosate, the surfactant being its primary toxic component. Furthermore, the observed cardiotonic effects, likely resulting from increased intracellular calcium in myocytes, are consistent with findings from other case reports. No adverse effects of lipid emulsions were reported in this study [26].

### Prospective uncontrolled study

A multicenter prospective uncontrolled study conducted in 2016 by Mithani et al. included patients identified through poison control center calls. Inclusion criteria consisted of exposure to a lipophilic cardiotoxic substance, hemodynamic instability, and failure of conventional therapies to stabilize the patient. All patients received ILE therapy as an antidote. The primary outcome was hemodynamic stabilization, measured by a minimum 10 mmHg increase in MAP within the first hour after ILE administration. A total of 36 patients were enrolled, of whom 25 survived. When patients had the last available single MAP value carried forward to 1 h, the mean change in MAP was 17.22 mmHg (*n* = 23; median = 13.33; *p* = 0.044 for one-sided *t*-test assessing MAP ≥ 10 mm Hg). Thirteen (56.6%) of these 23 patients had at least a 10 mmHg increase in MAP at 1 h after ILE administration (*p* = 0.677). This mean change in MAP was highly influenced by one extreme value for one patient (MAP increased by 76.7 mm Hg at 43 min after ILE administration); without this patient’s result, the mean change in MAP was 14.52 mm Hg at 1 h and was no longer statistically significant (median = 11.67; *p* = 0.085).

Further analysis of survivors’ subgroups revealed no statistically significant increase in MAP within the first hour (mean change = 9.83 mmHg; 95% CI − 4.34–23.99; *n* = 25; *p* = 0.180). The study ultimately failed to demonstrate a significant effect of lipid emulsions on the primary outcome of hemodynamic stabilization. Due to the absence of a control group, the study could not assess secondary outcomes such as mortality or hospital length of stay.

Regarding side effects, the study reported disturbances in laboratory parameters but did not systematically investigate or report other potential adverse effects attributable to ILE. The absence of a control group and limited methodological rigor restrict the study’s ability to draw firm conclusions about the efficacy or safety of ILE in this context [27] (Table 1).

**Table 1:** Review of recent data

Study	Type	Toxic agent	Population	Intervention	Control	Primary outcome	Significant findings	Adverse effects
Gil et al. (2010–2012)	Prospective cohort	Glyphosate poisoning	64 patients	Lipid Emulsion Therapy	Matched control group	Hypotension, arrhythmias	Reduced hypotension (0% vs. 40%), reduced arrhythmias (0% vs. 22.7%)	None reported
Taftachi et al. [18]	RCT	Non-local anesthetic drug poisoning	30 patients	Lipid Emulsion Therapy	Standard care	GCS improvement, extubation time	Significant improvement in GCS (*P* = 0.048)	None reported
Majeed et al. (2012–2014)	RCT	Organophosphate poisoning	100 patients	Lipid Emulsion Therapy	Standard care	GCS, MAP, ventilation requirements, ICU stay, mortality	Improved GCS, MAP, reduced ventilation, ICU stay, lower mortality	None reported
Elabdeen et al. (2016–2017)	RCT	Aluminum phosphide poisoning	50 patients	Lipid Emulsion Therapy	Standard care	Mortality, intubation and ventilation requirements	Lower need for ventilation (36% vs. 92%)	None reported
Mithani et al. [27]	Prospective uncontrolled study	Lipophilic cardiotoxic substances	36 patients	Lipid Emulsion Therapy	Uncontrolled	Hemodynamic stabilization (MAP increase)	No significant MAP improvement	Disturbances in lab parameters
Kazemifar et al. (2017–2018)	RCT	Tramadol poisoning	80 patients	Lipid Emulsion Therapy	Standard care	Seizure occurrence	No seizures in intervention group (*p* < 0.001)	None reported
Yu et al. [16]	Meta-analysis	Organophosphate Poisoning	630 patients (7 RCTs)	Lipid Emulsion Therapy	Standard care	Cure rate, mortality	Higher cure rate, reduced mortality (OR = 0.31, *p* = 0.009)	None reported
Behnoush (2019–2021)	RCT	Tramadol poisoning	120 patients	Lipid Emulsion Therapy	0.9% saline	GCS, hospital stay, seizure rates	Higher GCS (12 vs. 10 ; *p* = 0.03), shorter hospital stay (*p* < 0.01), lower seizure rates (*p* < 0.01)	None reported
Basiouny et al. (2019–2020)	RCT	Antipsychotic poisoning	40 patients	Lipid Emulsion Therapy	Standard care	GCS, ECG changes, ICU admissions	Higher GCS, reduced ICU admissions, improved ECG findings	None reported
Elgazzar et al. (2019–2020)	RCT	Clozapine poisoning	40 patients	Lipid Emulsion Therapy	Standard care	GCS, QTc prolongation, hospital stay	Higher GCS, shorter hospital stay, lower QTc prolongation	None reported
Chhabria et al. [25]	Prospective cohort	Organophosphate poisoning	40 patients	Lipid Emulsion Therapy	Historical control group	Morbidity, mortality, adverse effects	Shorter ventilation, hospital stay, hypernatremia resolution	None reported
Pannu et al. [24]	RCT	Organophosphate poisoning	45 patients	Lipid Emulsion Therapy	Standard care	Atropine dose, hemodynamic, hospital stay, mortality	No significant benefits observed	None observed

### Registries and literature review

The LIPAEMIC report is a prospective study conducted by Cave et al. from 2009 to 2012 which allowed hospital institutions to submit case report forms for intoxicated patients treated with ILEs via a dedicated website. A total of 48 patients were included, with 38 cases involving toxicity from lipophilic substances other than local anesthetics. In three cases of non-LAST, survival was attributed to ILEs administration. Among 30 non-local anesthetic cases, ILEs were primarily used to treat altered consciousness. In 26 patients, consciousness improved within 30 min post-administration, with the median GCS score increasing from 4/15 to 8/15. Additionally, eight cases involving hemodynamic instability showed an increase in MAP from 70 to 90 mmHg following ILEs administration. However, the authors noted that the improvements could not be conclusively linked to ILEs due to confounding factors, such as concurrent treatments and the lack of a control group. Adverse effects potentially attributable to ILEs included a severe bronchospastic reaction, hyperamylasemia, and interference with laboratory analyses [7].

A retrospective study conducted in 2013 by Presley et al., focused on pediatric patients under 18 years of age who experienced toxicity from local anesthetics (7 patients) or other lipophilic substances, such as amitriptyline, diltiazem, bupropion, dosulepin, lamotrigine, quetiapine, and verapamil. The study included 14 cases, with improvement observed in 13. Recovery was characterized by one or more of the following: restoration of consciousness, normalization of electrocardiogram, and hemodynamic stabilization. For cases of LAST, improvement occurred within seconds to minutes after ILEs administration, while for non-LAST cases, improvement was noted within minutes to hours. One 15-year-old patient with severe verapamil intoxication did not benefit from ILEs therapy, and a single case of acute pancreatitis was reported, attributed to hypertriglyceridemia following ILEs administration [28].

In a 2011 literature review by Cave et al., 42 clinical cases involving ILEs in toxicology were documented. Nineteen cases involved LAST, while the remaining 23 related to other drug classes. ILEs were primarily used in cases of hemodynamic instability or neurological deterioration. The authors applied the “WHO-UMC system for standardized case causality” to assess the likelihood that clinical improvements were attributable to ILEs. Using the Landis and Koch method, moderate inter-rater agreement was achieved. In one unanimous case of refractory cardiac arrest due to bupropion, definite improvement was linked to ILEs. For 10 additional cases (8 involving LAST and 2 involving other substances), probable clinical improvement was reported. Divergent opinions among the authors precluded conclusions in the remaining cases. Adverse effects included one case of hyperamylasemia and one of acute respiratory distress syndrome (ARDS), though a direct causal link with ILEs could not be established [29].

Levine et al. [30] conducted a systematic review evaluating the use of ILEs in the management of acute poisoning with non-local anesthetic agents. The review included 203 studies, combining human data (mainly case reports and case series) and animal studies. The quality of evidence was generally assessed as low to very low. Most randomized studies were conducted in animal models, whereas human data relied almost exclusively on anecdotal reports. Furthermore, the frequent use of multiple concurrent treatments in case reports limits the ability to isolate the specific effect of ILE. Considerable heterogeneity was also noted in the amount and formulation of lipid administered, the timing relative to other interventions, and the use of bolus versus continuous infusion—highlighting the lack of standardization in clinical practice [30].

A 2019 literature review by Panetta et al. analyzed 298 clinical cases of ILE use. Notably, 80% of cases did not involve local anesthetic intoxication. Clinical improvement following ILEs administration was reported in 96% of LAST cases and 66% of cases involving other substances. Interestingly, clinical improvement was observed in 62% of cases involving non-lipophilic substances. Acute pancreatitis and interference with laboratory analyses were the most frequently reported side effects. While acute pancreatitis is the most commonly described complication, its true incidence remains unclear due to significant variability across case series. Other less common adverse effects included bronchospasm, obstruction of continuous venovenous hemofiltration (CVVH) circuits, and ARDS [9].

### Summary of efficacy

The available data on the efficacy of ILEs in toxicology remains limited, with RCTs demonstrating partial effectiveness in specific cases of poisoning. Studies have reported some success in managing toxicities caused by tramadol, clozapine, glyphosate, and aluminum phosphide [19–21, 23]. For organophosphate poisoning, ILEs appear to improve prognosis and liver function, although the results across studies remain contradictory [16, 17, 25]. Conversely, a Canadian uncontrolled prospective cohort study failed to demonstrate the efficacy of ILEs in patients with hemodynamic instability [27].

Another uncontrolled cohort study suggested potential benefits of ILEs in reducing mortality in LAST and improving the GCS in poisonings involving lipophilic substances. However, no hemodynamic improvement was observed in cases unrelated to local anesthetics [7].

Retrospective studies based on case reports are particularly prone to significant biases. Firstly, there is a tendency for authors to publish cases where ILE therapy appears to have been effective, leading to publication bias. Secondly, observed clinical improvements, such as better GCS scores or hemodynamic stabilization, are not always attributable to ILEs and may instead result from concurrent interventions, such as fluid resuscitation, vasopressor administration, or the natural elimination of the toxin.

To address these biases, one retrospective study analyzed 42 case reports and found no definitive consensus except in cases involving local anesthetics [29]. A larger retrospective review of 215 case reports noted clinical improvement in 96% of LAST cases and 66% of cases involving other substances. However, these findings did not account for confounding factors or publication biases, highlighting the need for more rigorous and controlled studies to establish the efficacy of ILEs in toxicology [9].

## Side effects

The administration of ILEs in parenteral nutrition has been associated with serious adverse events, particularly in neonates. Cases of pediatric acute respiratory distress syndrome caused by fat embolisms have been documented. Other reported symptoms include headaches, jaundice, hepatosplenomegaly, and spontaneous bleeding. Collectively, these manifestations form what is known as lipid overload syndrome, a rare complication of prolonged ILE administration in parenteral nutrition [31].

Another significant potential adverse effect of ILEs is acute pancreatitis, which can occur when triglyceride levels exceed 900 mg/dL. At this threshold, triglyceride-rich chylomicrons circulate in the blood, potentially obstructing pancreatic capillaries and triggering inflammation. Several cases of acute pancreatitis following ILE administration have been documented [28, 32]. Moreover, elevated triglyceride levels can interfere with laboratory analyses, leading to inaccuracies in hematological and biochemical test results, even when triglyceride levels are not excessively high. This issue can be mitigated by obtaining blood samples before ILE administration or by performing brief high-speed centrifugation on the serum [33].

The interactions between lipid emulsions and other medications are not yet fully understood. However, no adverse events have been reported during co-administration with atropine, bicarbonate, or calcium. Nonetheless, caution is advised when combining ILEs with epinephrine during lipid resuscitation in bupivacaine overdose. Animal studies suggest that epinephrine-induced hyperlactatemia and acidosis may reduce the efficacy of lipid therapy. Based on these findings, the American Society of Regional Anesthesia (ASRA) recommends limiting epinephrine boluses to 1 mcg/kg during the treatment of LAST (Grade 2 A) [34]. Additionally, ILEs might enhance gastrointestinal absorption of lipophilic drugs, potentially exacerbating poisoning. For instance, studies have indicated that ILE administration prior to the onset of cardiovascular toxicity could increase the absorption of orally ingested lipophilic drugs such as amitriptyline, although the exact molecular mechanisms remain unclear [1, 35].

Another less frequently reported but clinically significant complication is the occlusion of extracorporeal circulation circuits, including CVVH, extracorporeal membrane oxygenation (ECMO), and CytoSorb. Nevertheless, this obstruction may be dependent on the volume of lipid emulsion administered. Adverse effects appear to be proportional to both the infusion rate and the total dose received. Hypersensitivity and allergic reactions have also been described as potential risks associated with ILE administration [9, 36–39].

Some of these side effects were highlighted in a retrospective study conducted by Levine et al. across two U.S. university hospitals. Over a 13-year period, 9 patients received ILEs as an antidote, and 6 experienced adverse effects. Among them, two patients developed acute pancreatitis, with triglyceride levels peaking at 8611 mg/dL and 3648 mg/dL, respectively. Laboratory interferences rendered blood test results unusable in four patients, despite attempts at centrifugation. Additionally, three patients developed ARDS, although it remains difficult to establish a direct causal relationship with ILE use [32].

At this stage, estimating the true prevalence of ILE-related adverse effects in toxicology remains challenging. Among the prospective studies included in this review, only one case of a severe bronchospastic reaction was reported, while other adverse events were generally mild, such as laboratory interferences and elevated serum amylase (hyperamylasemia). In contrast, the retrospective study by Levine et al. reported a higher incidence of serious complications, with 2 out of 9 patients experiencing severe adverse events. However, data from case reports remain too limited and heterogeneous to allow for a reliable estimation of the true incidence of ILE-associated side effects.

Overall, while ILEs are generally considered safe, adverse events—although uncommon—can be clinically significant. The most serious complications are typically related to the volume and rate of infusion and may include fat overload syndrome, pancreatitis, and mechanical obstruction of extracorporeal circuits. To minimize these risks, it is recommended to limit the total ILE dose to approximately 10–12 mL/kg. Additionally, clinicians should consider monitoring serum triglyceride levels and centrifuging blood samples briefly at high speed to reduce analytical interference in laboratory testing [38].

Larger prospective studies are warranted to better characterize the safety profile of ILEs and to identify patient populations at greater risk for adverse effects.

## Recommendations

According to the 2021 ERC guidelines, ILEs are recommended as a first-line treatment for LAST. Additionally, their use should be considered in cases of intoxication involving beta-blockers, calcium channel blockers, and tricyclic antidepressants [40].

In the United States, the AACT published expert recommendations in 2016 to establish a framework for ILE therapy. Based on the available literature, experts assessed both the level of evidence and the strength of recommendations. For bupivacaine poisoning, the level of evidence remains very low, yet the recommendation is strong (1D) in cases of bupivacaine-induced cardiac arrest. However, in 2018, the Third American Society of Regional Anesthesia and Pain Medicine Practice Advisory on Local Anesthetic Systemic Toxicity provided a strong recommendation with higher evidence (Level IB). In cases of severe poisoning without cardiac arrest, ILEs are recommended by the AACT as part of the therapeutic arsenal, though their priority within treatment strategies is not specified (2D).

For amitriptyline and bupropion intoxications, the AACT recommends ILE use in life-threatening cases, but only as a last-resort intervention (2D). For other substances, the AACT refrains from making specific recommendations and instead suggests either non-use or a neutral stance. For example, in cases of severe poisoning with lipophilic beta-blockers or calcium channel blockers (e.g., verapamil and diltiazem), the AACT advises against using ILEs as a first-line intervention (2D).

The limited role of ILEs, according to the AACT, is primarily attributed to two factors. First, the lack of high-quality evidence demonstrating clear efficacy. Second, potential interactions with standard resuscitation methods, including occlusion of extracorporeal circulation circuits, possible interactions with insulin-glucose or glucagon therapy, and increased intestinal toxin absorption—a theoretical effect observed in animal studies [41].

Also in 2016, the American College of Medical Toxicology (ACMT) stated that there are no mandatory standards governing the use or non-use of ILEs. However, in cases of severe hemodynamic instability or toxicity from highly lipophilic substances, ILEs are considered a reasonable therapeutic option. The ACMT-recommended administration protocol is as follows:

- Bolus: A 20% lipid emulsion is administered as a 1.5 mL/kg bolus over 2 to 3 min.
- Second bolus: If the initial response is unsatisfactory, a second bolus may be administered.
- Infusion: Immediately following the bolus, a 20% lipid emulsion is infused at 0.25 mL/kg/min. After 3 min, the response should be evaluated. If significant improvement is observed, the infusion rate can be reduced to 0.025 mL/kg/min (one-tenth of the initial rate).

To minimize adverse effects from high cumulative lipid doses, the maximum recommended dose is 10 mL/kg. Blood pressure, heart rate, and other hemodynamic parameters should be recorded every 15 min during the infusion [42]. Moreover, blood triglycerides could be followed to avoid levels that are unsafe [43].

In 2023, the AHA updated its guidelines for the management of cardiac arrest due to life-threatening poisoning. Concerning lipid emulsions, the recommendation remains unchanged: “The use of 20% intravenous lipid emulsion can be efficacious in the resuscitation of life-threatening local anesthetic toxicity, especially from bupivacaine” [44].

In cases of severe poisoning with lipophilic β-blockers or calcium channel blockers, current evidence-based guidelines from AHA advise against the routine use of ILEs. Reported adverse effects include clogging of VA-ECMO filters, pancreatitis, and sudden cardiovascular collapse following ILE administration in patients with oral β-blocker overdose. Similarly, experimental and clinical data suggest that ILEs may increase the gastrointestinal absorption of lipophilic drugs, potentially exacerbating toxicity in cases of oral calcium channel blocker overdose.

For sodium channel blockers—such as tricyclic antidepressants, lamotrigine, chloroquine, cocaine, and venlafaxine—the AHA notes that it may be reasonable to consider ILEs in patients with life-threatening poisoning refractory to conventional therapies.

## Conclusion

Intravenous lipid emulsions were initially shown to be effective in the treatment of severe bupivacaine poisoning, a highly lipophilic agent. This led to the early development of the “lipid sink” hypothesis [32]. Following these initial findings, numerous case reports have described the use of ILEs in poisonings involving other lipophilic substances [10].

Subsequent research has led to a more nuanced understanding of their mechanism of action, now considered multimodal. In addition to the lipid sink, a lipid shuttle mechanism has been proposed, promoting the redistribution of lipophilic toxins toward detoxification organs such as the liver and skeletal muscle.

Moreover, ILEs are thought to exert a cardiotonic effect through several complementary mechanisms:

- Intravascular volume expansion,
- Inhibition of endothelial-derived NO release,
- Support of mitochondrial function,
- Stabilization of cell membranes, including reversal of sodium channel blockade, and.
- Phosphorylation of glycogen synthase kinase-3β (GSK-3β), which may reduce myocardial injury.

However, while an antidotal effect has been suggested for other substances, its efficacy remains less definitive than for bupivacaine [35]. Analysis of case reports suggests clinical improvement in 96% of bupivacaine poisoning cases, compared to 66% for other substances [8]. However, these findings are largely derived from case studies, which are subject to significant biases, resulting in low-quality evidence. Based on these data, the AACT issued 2016 guidelines strongly recommending ILEs for bupivacaine poisoning, while advising caution for other lipophilic substances, suggesting their use only as a last resort.

Since these guidelines were published, several RCTs have investigated the use of ILEs for other lipophilic toxins, including tramadol, clozapine, organophosphates, and glyphosate surfactants. These studies suggest that ILEs may improve neurological symptoms, reduce hospitalization duration, and decrease the need for mechanical ventilation. Furthermore, adverse effects appear infrequent when recommended doses are used. However, no significant mortality benefit has been demonstrated, likely due to small sample sizes and the absence of deaths in the studied groups. Additionally, no RCTs have specifically evaluated ILEs in severe poisonings involving beta-blockers, calcium channel blockers, and tricyclic antidepressants, nor have they included patients with severe hemodynamic instability caused by intoxication.

To optimize the use of ILEs across all lipophilic substances, additional data are required to tailor their administration to specific toxins, taking into account the unique pharmacokinetic and pharmacodynamic profiles of each compound. Furthermore, the true incidence of ILE-related adverse effects, such as acute pancreatitis, seems to be low but remains uncertain. To minimize the risk of such complications, clinicians should limit both the rate and volume of infusion and monitor blood triglyceride levels.

Despite the lack of high-quality data, lipid emulsions should undeniably remain part of the therapeutic arsenal. The large number of reported cases provides a strong basis for their continued use, although higher-quality studies are needed to further substantiate their efficacy and safety. Conducting large-scale RCTs remains challenging, particularly due to ethical concerns about enrolling hemodynamically unstable patients, such as those poisoned by beta-blockers or calcium channel blockers. In our opinion, establishing new prospective registries, similar to the “LIPAEMIC report” could supplement existing data and potentially lay the groundwork for larger clinical trials [6].

## Data Availability

Not applicable.

## Abbreviations

AACT: American Academy of Clinical Toxicology
ACMT: American College of Medical Toxicology
AHA: American Heart Association
ARDS: Acute Respiratory Distress Syndrome
ASRA: American Society of Regional Anesthesia
ATP: Adenosine triphosphate
CVVH: Continuous venovenous hemofiltration
ECMO: Extracorporeal membrane oxygenation
ERC: European Resuscitation Council
GCS: Glasgow Coma Scale
ILE: Intravenous lipid emulsion
LAST: Local anesthetic systemic toxicity
MAP: Mean arterial pressure
NO: Nitric oxide
OP: Organophosphate
RCT: Randomised controlled trial
ROS: Reactive oxygen species
